# Varietal responses of root characteristics to low nitrogen application explain the differing nitrogen uptake and grain yield in two rice varieties

**DOI:** 10.3389/fpls.2023.1244281

**Published:** 2023-08-03

**Authors:** Lei Liu, Kehui Cui, Xiaoli Qi, Yu Wu, Jianliang Huang, Shaobing Peng

**Affiliations:** ^1^ National Key Laboratory of Crop Genetic Improvement, Wuhan, Hubei, China; ^2^ Key Laboratory of Crop Ecophysiology and Farming System in the Middle Reaches of the Yangtze River, Ministry of Agriculture and Rural Affairs, Wuhan, Hubei, China; ^3^ College of Plant Science and Technology, Huazhong Agricultural University, Wuhan, Hubei, China

**Keywords:** apoplasmic pathway, ammonium transporter genes, lignification, low nitrogen application rate, nitrogen uptake, rice (*Oryza sativa* L.), root characteristics

## Abstract

Rice root characteristics are tightly associated with high-efficient nitrogen uptake. To understand the relationship of root plastic responses with nitrogen uptake when reducing nitrogen application for green rice production, a hydroponic experiment and a soil pot experiment were conducted under high (HN) and low (LN) nitrogen applications, using two rice (*Oryza sativa* L.) varieties, NK57 and YD6, three nitrogen absorption traits (total nitrogen accumulation, net NH_4_^+^ influx on root surface, nitrogen uptake *via* apoplasmic pathway) and root characteristics were investigated. In comparison with HN, LN significantly reduced nitrogen absorption and grain yield in both varieties. Concomitantly, there was a decrease in total root length, root surface area, root number, root volume, and root cortical area under LN, while single root length, root aerenchyma area, and root lignin content increased. The expression of *OsAMT1;1* and *OsAMT1;2* down-regulated in both varieties. The findings revealed that YD6 had smaller reduction degree for the three nitrogen absorption traits and grain yield, accompanied by smaller reduction degree in total root length, root surface area, root cortical area, and expression of the two genes under LN. These root characteristics were significantly and positively correlated with the three nitrogen absorption traits and grain yield, especially under LN. These results indicate that a large root system, lower reduction degree in several root characters, and high expression of *OsAMT* genes in YD6 explains its high nitrogen accumulation and grain yield under reduced nitrogen application. The study may provide rationale for developing varieties with low nitrogen fertilizer requirements for enabling green rice production.

## Introduction

China accounts for about 30% of the total global nitrogen fertilizer consumption. However, crop plants only use 40%-50% of externally applied nitrogen, resulting in inefficient nitrogen utilization and exerting huge pressure on natural ecological environments (Peng et al., 2010; Lassaletta et al., 2016). Therefore, improvement in nitrogen uptake and utilization has been attracting attention, especially in green production with lower fertilizer inputs (Cui et al., 2018). For improving nitrogen utilization, it is necessary to boost nitrogen uptake leading to more dry matter and grain yield even under less nitrogen supply (Shi et al., 2010; Chen et al., 2020). As plant roots are the main organ for acquiring soil resources and are closely linked to efficient nitrogen uptake, adequately exploring and utilizing root characteristics is an effective way to enhance crop nitrogen uptake (Lynch, 2019).

The crop nitrogen uptake and use are influenced by root morphological and anatomical characteristics (York et al., 2015; Wu et al., 2019). Previous studies have shown that the total root length, root surface area, root volume, and root number are the key root morphological traits that positively affect nitrogen uptake in rice (Wei et al., 2018; Chen et al., 2020). Hence, rice varieties with high nitrogen uptake efficiency often have large root systems (Ju et al., 2015; Wei et al., 2018; Chen et al., 2020). Additionally, maize varieties with high nitrogen uptake efficiency had large root diameters and more xylem vessels (York et al., 2015; Yang et al., 2019; Schneider et al., 2021), and thick absorptive roots showed high nitrogen uptake capacity (Zhang et al., 2020). When the same nitrogen fertilizer rate was applied, recent rice varieties often exhibit larger root diameters, better growth performance, and higher grain yields as compared to old varieties (Liu et al., 2014). Therefore, these root traits are tightly associated with nitrogen uptake in rice plants.

The apoplasmic pathway and cell-to-cell (symplastic and transcellular) pathway are the two parallel pathways for water and nutrient transport in plant roots (Barberon and Geldner, 2014). The Casparian bands and phellem often act as apoplasmic barriers (Barberon and Geldner, 2014; Tao et al., 2017). The enhanced lignin deposition in the root cell wall induced by salt and hypoxia stress accelerated apoplasmic barrier formation, which decreased root hydraulic conductance and restricts water and ions transport *via* the apoplasmic pathway in plant roots (Ranathunge et al., 2016; Tao et al., 2017; Huang et al., 2019). Similarly, the strong lignified apoplasmic barriers significantly reduced the permeability of NH_4_^+^ in rice seedling roots, which was detrimental to nitrogen uptake (Ranathunge et al., 2016). Additionally, root cortical aerenchyma (RCA) also functions as an apoplasmic barrier to inhibit the transport of water and ions in roots (Yang et al., 2012; Hu et al., 2014; Ren et al., 2015; Huang et al., 2019; Gao et al., 2020). Under drought stress, the high RCA formation decreased water uptake *via* the apoplasmic pathway in rice seedlings (Xu et al., 2017). Similarly, the phosphorus, calcium, and cadmium accumulations and transport *via* the apoplasmic pathway also dropped significantly in crop genotypes with high RCA as compared to low RCA (Hu et al., 2014; Huang et al., 2019). Therefore, the enhanced formation of lignified apoplastic barrier and RCA might not be beneficial for nitrogen uptake *via* the apoplasmic pathway in rice roots. Methodologically, trisodium-8-hydroxy-1,3,6-pyrenetrisulphonic acid (PTS), as a water-soluble fluorescent indicator cannot pass through the cell membrane or adhere to the cell wall (Vesk et al., 2000) and is often used as a tracer in simulating apoplasmic transport (Yadav et al., 1996; Tao et al., 2017).

Ammonium transporters viz. *AMT1;1*, *AMT1;2*, and *AMT1;3* account for most of the NH_4_^+^ uptake by rice roots (Yuan et al., 2007; Jiao et al., 2020). Transgenic rice seedlings with over-expression of *OsAMT1;1*, *OsAMT1;2*, and *OsAMT1;3* in roots exhibited higher NH_4_^+^ uptake (Ranathunge et al., 2014; Ferreira et al., 2015; Lee et al., 2020). The *AMT1;2* mediated NH_4_^+^ uptake *via* the apoplasmic pathway, and hence its function loss sharply reduced the NH_4_^+^ uptake capacity in *Arabidopsis* roots (Yuan et al., 2007). The two genes (*OsAMT1;1* and *OsAMT1;2*) were mainly expressed in the root epidermis and stele (Sonada et al., 2003; Li et al., 2016), which indicated that the two genes may also mediate the NH_4_^+^ uptake *via* apoplasmic pathway in rice plants (Yuan et al., 2007). On the other hand, high expression of ammonium transporter genes increased plant growth rate, and sugar and starch production. Rice plants with over-expression of ammonium transporter genes produced more grain yield and dry matter at the same nitrogen application (Ranathunge et al., 2014). Therefore, increasing the expression of nitrogen transport-related genes in roots is one of the approaches to enhance nitrogen uptake and use efficiency in rice.

Root traits are often regulated by varying nitrogen levels. Severe nitrogen deficiency significantly reduced total root length, root surface, and root volume in plants (Giehl and von Wirén, 2014). However, rice varieties with high nitrogen uptake maintained higher performances of root traits (such as root biomass, root length, root surface, and root volume) under LN (Shi et al., 2010; Ju et al., 2015). LN promoted crop root elongation (Shi et al., 2010; Gao et al., 2015), and a long root system is an ideal character to capture more nitrogen in the deep soil layer (Arai-Sanoh et al., 2015). Additionally, low nitrogen application induced RCA formation and increased root lignin content, which was not beneficial for water, cadmium, and nickel uptake (Kovaáčik et al., 2011; Ren et al., 2015). On the other hand, low nitrogen supply significantly downregulated the expression of *OsAMT1;1* and *OsAMT1;2* (Sonada et al., 2003) and significantly decreased NH_4_^+^ concentrations in root apoplasmic fluid (Yuan et al., 2007). Rice varieties with high nitrogen uptake exhibited high expression of *OsAMT1;1 OsAMT1;3* genes under low nitrogen stress (Shi et al., 2010; Gaur et al., 2012), and low NH_4_^+^ treatment enhanced the expression of *OsAMT1;3* in rice roots (Ferreira et al., 2015). These reports totally suggest that the high expression of *OsAMT* genes may be propitious to NH_4_^+^ uptake and transport *via* apoplasmic pathway in rice plants under low nitrogen supply. Generally, the responses of root morphological, anatomical, and physiological traits to nitrogen deficiency vary, depending on the crop, variety, and planting condition. Nevertheless, maintaining a large root system and enhancing the expression of *OsAMT* genes are beneficial for nitrogen uptake in rice plants exposed to low nitrogen conditions.

A major issue for China’s rice production is excessive nitrogen application. Zhang et al. (2018) reported overuse of nitrogen fertilizers in about 45% of rice fields in China, resulting in serious nitrogen loss and environmental challenges. In order to meet continued yield increase and quality improvement, super rice variety should possess high nutrient efficiency, promising to greatly reduce the consumption of chemical fertilizers, green rice production through the reduction in fertilizer usage has recently attracted a lot of attention (Zhang, 2007). In this study, two rice varieties with different performances in root characteristics and nitrogen uptake were used to investigate responses of root characteristics and NH_4_^+^ transport pathway under LN. The mechanism for efficient nitrogen uptake when nitrogen fertilizer application is reduced was also elucidated.

## Materials and methods

### Experiment 1 (hydroponic experiment with low and high nitrogen treatments)

The hydroponic experiment was conducted during the rice growth season in 2020 at the experimental station of Huazhong Agricultural University, Wuhan, China (30°29’ N, 114°22’ E), using two rice (*Oryza sativa* L.) varieties, Nonken 57 (NK57, Japonica rice) and Yangdao 6 (YD6, Indica rice). Compared with NK57, YD6 had significantly higher nitrogen uptake efficiency in the previous study (Zhu et al., 2015).

Rice seeds were disinfected in 10% H_2_O_2_ solution for 10 min and sown in plastic seeding trays with loam soil after breaking dormancy at 37 °C for 48 h. The seedlings with 3 visible leaves were transplanted to a 12 L plastic pot (25.5 cm in height, 24.4 cm in top diameter), eight seedlings were planted in each pot, which contained a hydroponic nutrient solution with two nitrogen levels i.e., high nitrogen concentration (40 mg L^-1^) and low nitrogen concentration (5 mg L^-1^) in the form of (NH_4_)_2_SO_4_. The other nutrient composition of the solutions followed the description by Yang et al. (2012): P as 10 mg L^-1^ NaH_2_PO_4_, K as 40 mg L^-1^ K_2_SO_4_, Ca as 40 mg L^-1^ CaCl_2_, Mg as 40 mg L^-1^ MgSO_4_, Fe as 2.0 mg L^-1^ Fe-EDTA, Mn as 0.5 mg L^-1^ MnCl_2_·4H_2_O, Mo as 0.05 mg L^-1^ (NH_4_)_6_Mo_7_O_24_·4H_2_O, B as 0.2 mg L^-1^ H_3_BO_3_, Zn as 0.01 mg L^-1^ ZnSO_4_·7H_2_O, Cu as 0.01 mg L^-1^ CuSO_4_·5H_2_O, and Si as 2.8 mg L^-1^ Na_2_SiO_3_·9H_2_O. Each nitrogen treatment and each variety were repeated three times, and nine pots were used for each of the four combinations across two varieties and two nitrogen treatments, six pots for each repetition. The nutrient solution was replaced every 4 days and the pH was adjusted to 5.50 ± 0.1 every day with 1 mol L^-1^ HCl or 1 mol L^-1^ NaOH. The uniform rice seedlings were sampled for measurements after 24 days following nitrogen treatment.

### Experiment 2 (soil pot experiment with low and high nitrogen treatments)

To investigate the relationships of root characteristics with rice grain yield formation and nitrogen accumulation at late growth stages, a soil pot experiment was conducted using the same varieties in the experiment 1. On the 24^th^ day after seed sowing, three uniform rice seedlings were transplanted to a 12 L plastic pot (25.5 cm in height, 24.4 cm in top diameter) filled with 10 kg sandy loam soil. The properties of the soil were as follows: 0.99 g total nitrogen kg^-1^, 113.7 mg exchangeable potassium kg^-1^, 8.11 mg Olsen phosphorous kg^-1^, pH 6.60, and 10.50 g organic matter kg^−1^. Two nitrogen application rates were used, high nitrogen (2.16 g nitrogen each pot) and low nitrogen (0.27 g nitrogen each pot) in the form of urea, which were spit-applied with three times, 40% of total nitrogen as basal fertilizer (before transplanting), 30% at tilling stage (12 days after transplanting) and 30% at panicle initiation stage. A total of 1.5 g phosphorous and 1.5 g potassium were applied for each pot as basal fertilizers in the form of calcium superphosphate and potassium chloride, respectively. The basal fertilizers in each pot were thoroughly mixed with the sandy loam soil before transplanting, the topdressing nitrogen fertilizers were dissolved in water and applied by an irrigating manner. Each nitrogen treatment was repeated thrice. During entire growth period, the rice plants were well watered with a shallow water layer of 1-2 cm, the weeds, pests and diseases were well controlled.

### Sampling and measurements

#### Measurement of dry weight and nitrogen content

At seedling stage in experiment 1, three uniform seedlings for each treatment were selected and washed, then were divided into roots, stems, and leaves, and placed in an oven at 105°C for 1 h to inactivate enzymes, followed by oven-drying at 80°C for determination of dry weights (**Table S1**). After dry weight determination, roots, stems and leaves were ground to a fine powder with a ball mill (MM301, Retsch GmbH, Haan, Germany), respectively.

At heading and maturity stages in experiment 2, three entire plants per treatment were washed with tap water and divided into panicles, roots, stems and leaves, these samples were oven-dried at 80°C to a constant weight and weighed. The oven-dried matters (roots, leaves, stems, panicles) in experiment 2 were also ground to a fine powder, respectively. Samples of 0.2 g were digested with H_2_SO_4_-H_2_O_2_ and nitrogen concentrations were measured using a Westco Smartchem 200 Discrete Analyzer (AMS-Alliance, France) according to the Kjeldahl method (Jimenez and Ladha, 1993), then nitrogen content and total nitrogen accumulation were calculated, respectively (**Table S2**).

#### Measurement of grain yield and its components

At maturity in experiment 2, three uniform plants per treatment were sampled. After roots were thoroughly washed with tap water, plants were divided into roots, panicles, stems and leaves. Effective panicles (filled grain number ≥5) were counted, then all spikelets of the panicles were manual-threshed. Unfilled grains (half-filled and empty grains) and filled grains were separated by immersing them in tap water. The plant parts (roots, leaves, stems, panicle rachides and unfilled grains, and filled grains) were oven-dried at 80°C for determination of dry weights (**Table S1**). The thousand grain weight (g), unfilled grains (half-filled and empty grains) and filled grains were counted. Grain yield (g plant^-1^), grain filling percentage (%), spikelets per panicle (No. panicle^-1^), total biomass (g plant^-1^), and harvest index (the ratio of grain yield to the total biomass, %) were calculated (**Table S3**).

#### Measurement of root morphological characteristics

Fresh roots from a single seedling were collected in experiment 1. At heading stage in experiment 2, the separated roots from a plant were washed with tap water. At each stage, two plants were used for each biological repeat. These root samples were evenly dispersed in a plastic tray with a thin water layer, respectively, and then scanned using an Epson v700 scanner at 300 dpi resolution. Then, the number of all adventitious roots of each plant was manually counted. The obtained images were used to determine total root length, root surface area, and root volume with the WinRhizo^®^ software (Regent Instruments, Quebec, Canada) (Chen et al., 2020). The single root length was calculated as the total root length divided by the root number.

#### Measurement of NH_4_^+^ influx on the root surface

The net NH_4_^+^ flux on the root surface was measured according to the method described by Sun et al. (2009). A single newly grown adventitious root (~8-12 cm) from an individual seedling in experiment 1 was selected to measure the net NH_4_^+^ influx rate using NMT (Non-invasive Micro-test Technology, Younger USA LLC, MA, USA). The root was equilibrated in the measuring solution (0.36 mM NH_4_Cl for LN-treated root, pH = 5.5; 2.86 mM NH_4_Cl for HN-treated root, pH = 5.5) for 10 min, and then transferred to a small petri dish (3 cm diameter) and immersed into 5 ml of measuring solution. To measure NH_4_^+^ flux, a microelectrode was automatically vibrated in the measuring solution between two points i.e., 5 μm, and 35 μm apart from the root surface along an axis perpendicular to the root. The NH_4_^+^ flux was recorded every 6 s for a total of 5 min using the imFluxes V3.0 software (NMT Physiolyzer^®^, Xuyue Company, Beijing, China), as presented in **Figure S1**. The single root was immediately used for anatomical observation. For each biological replicate, the measurement was repeated twice on two individual seedlings.

#### Measurement of root anatomical characteristics

The newly grown adventitious root (~8-12 cm) from an individual plant was selected to observe the root anatomical characteristics at heading stage in experiment 2. These adventitious roots in experiment 2 and the roots for measuring NH_4_^+^ flux in experiment 1 were cut into slices (~20-25 µm) at a distance of 2-2.5 cm from the root tip using a razor blade, then the excised thin root slice was placed on a glass slide with a drop of water for microscopic observation at 4 × magnification and photographed using Nikon Ti inverted fluorescence microscope (Nikon-Ti-SR, Japan). The obtained images were used to measure the living cortical area (μm^2^), RCA area (μm^2^), stele area (μm^2^), root diameter (μm), stele diameter (μm), and the number of xylem vessels using Image J software (ver. 1.39u; NIH, Bethesda, MD, USA, available at http://rsb.info.nih.gov/ij), according to the method reported by Hendel et al. (2021). The proportion of RCA (%) was referred to as the ratio of RCA area to the living cortical area.

#### Measurement of NH_4_^+^ uptake *via* apoplasmic pathway

The NH_4_^+^ uptake *via* the apoplasmic pathway was determined according to Tao et al. (2017) with minor modifications. A single rice seedling in experiment 1 was transplanted to a 1.2 L plastic pot (16 cm height × 10 cm top diameter) on the 24^th^ day after nitrogen treatment. The pot contained 10 mg L^-1^ of trisodium-8-hydroxy-1,3,6-pyrenetrisulphonic acid (PTS) in addition to the nutrient solution mentioned above and was covered with tinfoil and sealed with parafilm to prevent water loss by evaporation. The transplanted seedling was allowed to absorb PTS and NH_4_^+^ for 24 h. The volume of transpiration (L) during this period was measured by the weight difference method as described by Baxter et al. (2009). At the end of the period, PTS and NH_4_^+^ concentrations (mg L^-1^) in the nutrient solution were also measured, respectively. PTS concentration was measured using a Fluorescence Spectrophotometer (F-2700, HITACHI, Tokyo, Japan) at excitation and emission wavelengths of 403 and 510 nm, respectively. The NH_4_^+^ concentration was analyzed using a Westco Smartchem 200 Discrete Auto Analyzer (AMS-Alliance). Based on the difference in concentration, the amount of PTS and NH_4_^+^ uptake (mg) was calculated.

To obtain the transpiration stream concentration factor (TSCF), the amount of PTS and NH_4_^+^ per unit of transpiration stream (mg L^-1^) was computed by respectively dividing the PTS and NH_4_^+^ uptake by the volume of transpired water. Then, the calculated values were normalized with respect to the original PTS and NH_4_^+^ concentration in the nutrient solution, respectively. The corresponding normalized values were defined as TSCF for PTS and NH_4_^+^ (**Table S4**). The ratio of TSCF for PTS to TSCF for NH_4_^+^ (%) was considered as the NH_4_^+^ uptake *via* the apoplasmic pathway (Tao et al., 2017).

### Histologic location and measurement of root lignin

As described by Fu et al. (2014), root lignin histological staining and observation were performed. A razor blade was used to cut thin root slices from 12.5%, 25%, and 50% lengths of the newly formed adventitious roots (8-12 cm) from the root tip in experiment 1. These slices were soaked in 10 g L^-1^ phloroglucinol solution in 95% ethanol for 5 min and then rinsed with distilled water. The root slice was then acidified in 30% (w/w) hydrochloride solution for 1 min, and rinsed with distilled water again. After sealing by 50% (w/w) glycerol, the root slice was placed on a glass slide with a drop of water for microscopic observation and photographing using Nikon Ti inverted fluorescence microscope (Nikon-Ti-SR, Japan) at 4× magnification. The obtained images were used to observe the lignin histological distribution.

Root lignin content was measured using ultraviolet spectrophotometry, as described by Ren et al. (2015) with minor modifications. All roots of three rice seedlings were collected and oven-dried in experiment 1, followed by grinding to a fine powder using a ball mill (MM301, Retsch GmbH, Haan, Germany). The lignin was extracted from 10 mg of powder sample with 10 ml of 80% ethanol (v/v) under 50°C for 20 min, followed by centrifugation at 4024 g for 10 min, and removal of the supernatant. The extraction was repeated two times. The solid residue in the tube was collected and oven-dried to a constant weight at 80°C.

All of the oven-dried residues were transferred to a 2 ml plastic tube filled with 0.6 ml of 25% (v/v) acetyl bromide in acetic acid and 24 μl perchloric acid, which was then homogeneously mixed and incubated in an 80°C water bath for 60 min. The reaction mixture was added 0.6 ml of 2 mol L^-1^ NaOH to terminate the reaction and centrifuged at 8049 g for 10 min. Then 50 μl of supernatant was sampled and adjusted with acetic acid to 1.2 ml. The absorbance of the resulting solution was measured at 280 nm using an ultraviolet spectrophotometer (U-3900 UV-VIS spectrophotometer, Hitachi, Tokyo, Japan). Six dosages (from 1-6 mg) of pure lignin (Yuanye Bio-Technology Company, Shanghai, China) were sampled and performed in the same procedure to obtain a standard curve. The root lignin amount (mg) of the test powder sample was computed from the standard curve of lignin. The root lignin content (%) was expressed as the ratio of the lignin amount to the weight of the test sample.

### RNA extraction, reverse transcription, and real-time PCR

Full-length fresh roots (~8-12 cm) of five rice seedlings were used to extract total RNA in experiment 1, using Trizol Reagent (Sigma, USA) according to the manufacturer’s instructions. The concentration and purity were determined using Nanodrop 2000 spectrophotometer (Thermo Fisher Scientific, USA). After removing genomic DNA from RNA extract with DNase I (Invitrogen, USA), the first strand cDNA was synthesized with M-MLV reverse transcriptase (Invitrogen, USA) according to the manufacturer’s protocol. After a 10-fold dilution of the synthesized cDNA solution, quantitative real-time PCR was performed on ABI ViiATM7 real-time PCR system (Applied Biosystems, USA), using a Roche fast start universal SYBR Green master (Rox) kit (Roche, Switzerland). The procedure was 95°C for 2 min, followed by 45 cycles at 95°C for 10 s, 60°C for 10 s, and 72°C for 20 s. The housekeeping rice gene *Actin* was used as the internal control, and the expression analyses of ammonium transporter genes were carried out using the gene-specific primers listed in **Table S5** (Gaur et al., 2012) The relative quantification of gene expression was calculated using the 2^−ΔΔCT^ method (Livak and Schmittgen, 2001), and the average expression level of *OsAMT1;1* gene in YD6 across three biological replicates under LN was set as 1.

### Statistical analyses

The values for the investigated traits were expressed as means of three replicates, and were compared between the two nitrogen treatments for an identical variety and between the two varieties under an identical nitrogen treatment based on the least significant difference (LSD) test at a 5% probability level, using Statistix 9 software package (Analytical Software, Tallahassee, FL, USA). The Sigmaplot 10.0 software package (SPSS Inc., Chicago, IL, USA) was utilized for plotting and linear correlation analyses. In the study, the three traits (total nitrogen accumulation, net NH_4_^+^ influx, and NH_4_^+^ uptake *via* apoplasmic pathway) were considered as nitrogen absorption traits.

## Results

### Grain yield, dry matter, nitrogen, accumulation, and net NH_4_^+^ influx

As compared to HN, LN significantly decreased nitrogen uptake in both varieties (**Table 1**). In experiment 1, total nitrogen accumulation, net NH_4_^+^ influx on the root surface, and NH_4_^+^ uptake *via* apoplasmic pathway decreased by 75.2%, 73.1% and 90.5% in NK57, 71.2%, 66.3% and 84.3% in YD6 under LN in comparison to HN, respectively (**Table 1**; **Figure S1**). YD6 had higher performances in total nitrogen accumulation and net NH_4_^+^ influxes than NK57 under both HN and LN. The two varieties had similar NH_4_^+^ uptake *via* the apoplasmic pathway under HN, however, YD6 had significantly higher value than NK57 under LN (**Table 1**).

**Table 1:** The nitrogen absorption traits in YD6 and NK57 under low and high nitrogen applications at seedling stage (experimental 1) and heading and maturity stages (experiment 2).

N treatment	Variety	Experiment 1 (seeding stage)			Experiment 2 (heading and maturity stages)	
		TN	NI	NU	TN at heading stage	TN at maturity stage
		mg plant^-1^	pmol cm^-2^ s^-1^	%	mg plant^-1^	mg plant^-1^
LN	NK57	7.54 b	33.25 b	3.42 b	297 b	312 b
	YD6	9.76 b*	56.23 b*	5.98 b*	331 b*	391 b*
HN	NK57	30.44 a	123.59 a	35.83 a	643 a	661 a
	YD6	34.35 a*	166.62 a*	38.03 a	596 a	638 a

TN: total nitrogen accumulation; NI: net NH_4_^+^ influx, NU: NH_4_^+^ uptake via apoplasmic pathway, HN: high nitrogen application (40 mg L^-1^), LN: low nitrogen application (5 mg L^-1^); different lower-case letters within an identical column indicate significant difference between HN and LN at P < 0.05 (LSD test) for the same rice variety, * within an identical column indicates significant difference between YD6 and NK57 at P < 0.05 (LSD test) for the given nitrogen treatment.

In experiment 2, total nitrogen accumulations at heading and maturity stages decreased by 53.9% and 52.8% in NK57, 44.6% and 38.7% in YD6 under LN compared to HN (**Table 1**). No significant differences in total nitrogen accumulations were observed between the two varieties at heading and maturity stages under HN, however, YD6 exhibited higher total nitrogen accumulations than NK57 at the two stages under LN (**Table 1**).

Under both LN and HN in experiment 1, YD6 exhibited higher dry weights of roots, stems, leaves, and total dry weight than NK57 (**Tables 2**; **S1**; **Figure S2**), while no significant difference was observed in root, stem, and leaf nitrogen concentrations between YD6 and NK57 under HN and LN, respectively (**Table S2**).

**Table 2:** The dry weight and grain yield in NK57 and YD6 under low and high nitrogen applications at seedling and heading and maturity stages.

N treatment	Variety	Experiment 1 (seedling stage)		Experiment 2 (heading and maturity stages)				
		Root	Total	Root_HD_	Total_HD_	Root_M_	Total_M_	Grain yield
		g plant^-1^	g plant^-1^	g plant^-1^	g plant^-1^	g plant^-1^	g plant^-1^	g plant^-1^
LN	NK57	0.19 b	0.65 b	3.43 b	31.51 b	3.13 b	41.38 b	16.18 b
	YD6	0.24 b*	0.85 b*	4.15 b*	38.77 b*	3.52 b	51.63 b*	18.82 b*
HN	NK57	0.35 a	1.47 a	5.41 a	43.58 a	3.75 a	63.11 a	23.10 a
	YD6	0.42 a*	1.85 a*	5.96 a*	49.92 a*	3.97 a	68.57 a	24.60 a

Root_HD_: root dry weight at heading stage in experiment 2; Total_HD_: total dry weight at heading stage in experiment 2; Root_M_: root dry weight at maturity stage in experiment 2; Total_M_: total dry weight at maturity stage in experiment 2; HN: high nitrogen application (40 mg L^-1^), LN: low nitrogen application (5 mg L^-1^); different lower-case letters within an identical column indicate significant difference between HN and LN at P < 0.05 (LSD test) for the same rice variety; * within an identical column indicates significant difference between YD6 and NK57 at P < 0.05 (LSD test) for the given nitrogen treatment.

In experiment 2, root dry weight and total dry weight at heading stage decreased by 36.6% and 27.7% in NK57, 30.4% and 22.3% in YD6 under LN, compared to HN (**Table 2**). In addition, root dry weight, total dry weight and grain yield at maturity stage decreased by 16.5%, 34.4% and 30.0% in NK57, 11.8%, 24.7% and 23.5% in YD6 under LN in comparison with HN, respectively (**Table 2**). At heading stage, YD6 had higher root dry weight and total dry weight than NK57 under both LN and HN (**Table 2**). At maturity stage, YD6 exhibited the similar grain yield, total dry weight and grain nitrogen concentration with NK57 under HN, however, these three parameters were significantly higher in YD6 than those in NK57 under LN (**Table 2**; **Table S2**).

### Root morphological characteristics

**Figure 1:**
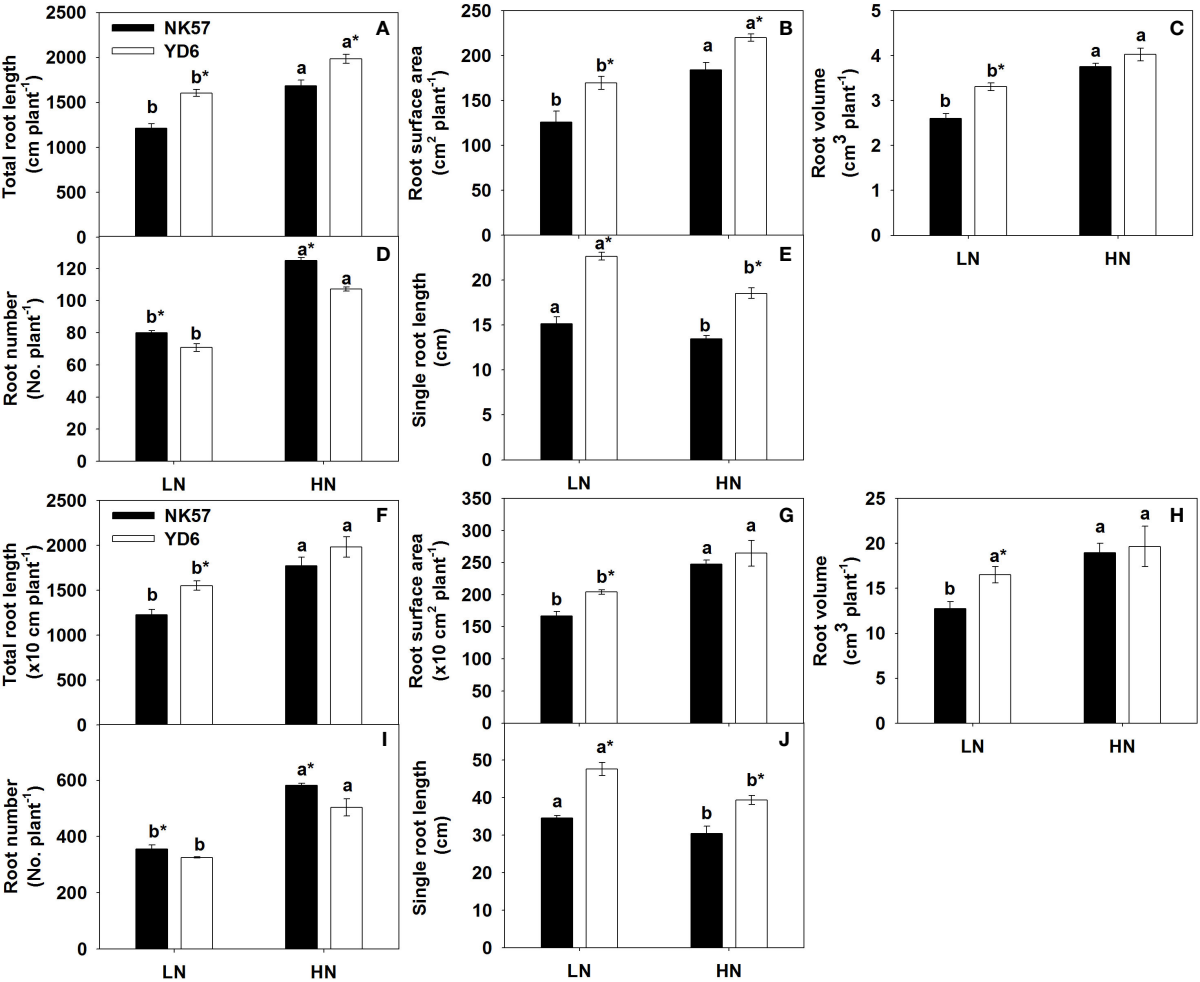
The root morphological characteristics in NK57 and YD6 under low and high nitrogen applications at seedling stage (experimental 1) and heading stage (experiment 2). HN: high nitrogen application (40 mg L^-1^), LN: low nitrogen application (5 mg L^-1^).Vertical bars indicate mean ± standard deviation (n=3). **(A-E)**: root morphological characteristics at seedling stage (experiment 1). **(F-J)**, root morphological characteristics at heading stage (experiment 2). Different lower-case letters on top of histograms denote significant difference between HN and LN at *P* < 0.05 (LSD test) for the same rice variety; * on top of histograms indicates significant difference between YD6 and NK57 at *P* < 0.05 (LSD test) for the given nitrogen treatment.

In NK57 and YD6, total root length, root surface area, root volume, and root number decreased at seedling stage (**Figures 1A–D**) and heading stage (**Figures 1F–I**) under LN, as compared to those under HN. Contrarily, LN significantly increased single root length at the two stages (**Figures 1E, J**; **S2**). YD6 exhibited larger phenotypic values in the five root traits at the two stages under LN, in comparison with NK57 (**Figure 1**). Additionally, in experiment 1, total root length, root surface area, root volume, and single root length of YD6 were 17.8%, 19.5%, 7.3%, 37.5% higher than those of NK57 under HN, respectively; however, the four parameters of YD6 were 32.0%, 34.8%, 27.2%, and 49.4% higher than those of NK57 under LN (**Figures 1A–C, E**). In experiment 2, no significant difference was observed in total root length, root surface area, root volume between NK57 and YD6 under HN, however, these three parameters were significantly higher in YD6 than those in NK57 under LN (**Figures 1F–H** **)**. The single root length in YD6 was 13.6% and 21.1% higher than that in NK57 under HN and LN, respectively (**Figure 1J**). In both experiments 1 and 2, YD6 had lower root number than NK57 under HN and LN (**Figures 1D, I**).

In NK57 and YD6, there was no significant difference in stele area, root diameter, stele diameter, and number of xylem vessels between LN and HN in experiment 1, respectively (**Table 3**; **Figure S3**). In experiment 2, compared to HN, LN significantly decreased root diameter and stele diameter in NK57, but had no effects on the two parameters in YD6 (**Table 3**). In both experiments 1 and 2, living cortical area under LN was similar to that under HN in YD6, however, LN significantly decreased the living cortical area in NK57. The proportion of RCA increased in NK57 under LN but not significant in YD6 in experiment 1, and increased in the two varieties in experiment 2 (**Table 3**). YD6 had higher living cortical area, stele area, root diameter, stele diameter, and number of xylem vessels than NK57 under both HN and LN in experiments 1 and 2 (**Table 3**; **Figure S3**). Most of these differences were significant.

**Table 3:** The root anatomical characteristics in NK57 and YD6 under low and high nitrogen applications at seedling and heading stages.

Nitrogen treatment	Variety	LCA	RCA	SA	RD	SD	XN
		×10^3^ μm^2^	%	×10^3^ μm^2^	μm	μm	No.
Experiment 1 (seedling stage)							
LN	NK57	471 b	12.97 a*	26 a	851 a	168 a	3.67 a
	YD6	711 a*	6.01 a	41 a*	1017 a*	210 a*	4.83 a*
HN	NK57	533 a	8.30 b*	27 a	873 a	169 a	4.00 a
	YD6	734 a*	4.25 a	40 a*	1018 a*	211 a*	4.67 a*
Experiment 2 (heading stage)							
LN	NK57	461 b	31.32 a*	23 a	820 b	156 b	4.25 a
	YD6	642 a*	22.87 a	29 a	950 a*	175 a	4.67 a*
HN	NK57	533 a	21.01 b	27 a	875 a	169 a	4.17 a
	YD6	647 a	15.82 b	30 a	960 a*	175 a	4.75 a*

HN: high nitrogen application (40 mg L^-1^), LN: low nitrogen application (5 mg L^-1^); LCA: living cortical area, RCA (%): the proportion of root aerenchyma, SA: stele area, RD: root diameter, SD: stele diameter, XN: number of xylem vessels, CCFN: cortical cell file number; lower-case letters within an identical column indicate significant difference between HN and LN at P < 0.05 for the same rice variety; * within an identical column indicates significant difference between YD6 and NK57 at P < 0.05 (LSD test) for the given nitrogen treatment.

### Root lignification

As compared to HN, enhanced lignin depositions were observed in root sclerenchyma, endodermis cells, and xylem in both the varieties under LN (**Figure S4A**). However, there was no visible difference between YD6 and NK57 under LN and HN (**Figure S4A**). Root lignin content increased by 24.7% in NK57 and 20.5% in YD6 under LN in comparison with those under HN (**Figure S4B**); however, the values showed no significant difference between two varieties under both LN and HN.

### The relative expression level of NH_4_^+^ transporter genes

**Figure 2:**
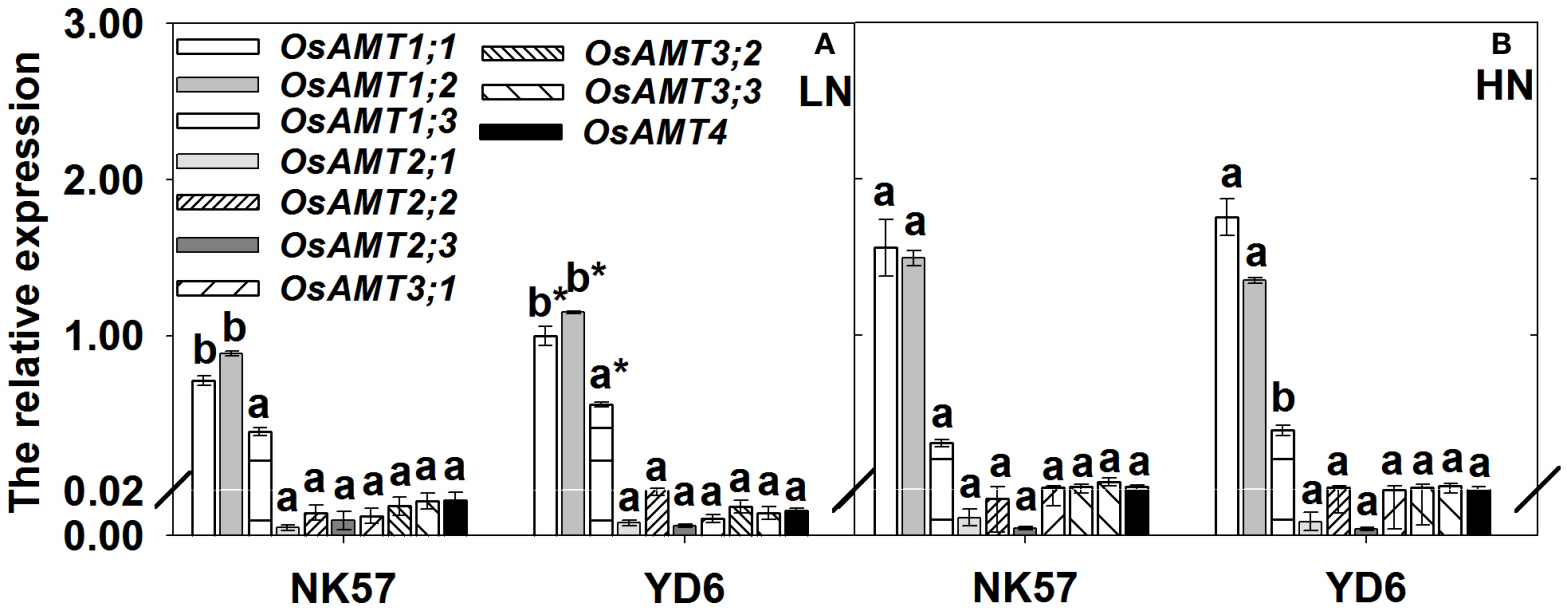
The relative expression level of ammonium transporter genes in YD6 and NK57 under low **(A)** and high **(B)** nitrogen applications at seedling stage (experimental 1). Vertical bars indicate mean ± standard deviation (n=3); The average expression level of *OsAMT1;1* gene of YD6 across three biological replicates under LN was set as 1. HN: high nitrogen application (40 mg L^-1^), LN: low nitrogen application (5 mg L^-1^). Different Lower-case letters on top of histograms denote significant difference between HN and LN at *P* < 0.05 (LSD test) for the same rice variety; * on top of histograms indicates significant difference between YD6 and NK57 at *P* < 0.05 (LSD test) for the given nitrogen treatment.

In NK57 and YD6, the expression level of *OsAMT1;1*, *OsAMT1;2* genes were significantly downregulated under LN (**Figures 2A, B**). Compared to NK57, YD6 exhibited significantly higher expression levels of *OsAMT1;1*, *OsAMT1;2*, *OsAMT1;3* genes under LN (**Figure 2A**). However, no significant difference was observed between NK57 and YD6 under HN (**Figure 2B**).

### Correlations of nitrogen absorption traits with root morphological characteristics

At seedling stage in experiment 1, total root length, root surface area, root volume and single root length were positively and significantly correlated with total nitrogen accumulation and net NH_4_^+^ influx under both LN and HN, however, root volume was not significantly correlated with the two nitrogen absorption traits under HN (**Table 4**). The four root traits were positively and significantly correlated with NH_4_^+^ uptake *via* apoplasmic pathway under LN. Root number were negatively and significantly correlated with the three nitrogen absorption traits but not with NH_4_^+^ uptake *via* apoplasmic pathway under LN. Similarly, the five root traits mentioned above were significantly correlated with total nitrogen accumulation at heading and maturity stages only under LN in experiment 2 (**Table 4**).

**Table 4:** Correlations of root morphological characteristics at seedling and heading stages with grain yield, total nitrogen accumulation, net NH_4_^+^ influx, and NH_4_^+^ uptake *via* apoplasmic pathway.

Traits	Nitrogen treatment	Total Root length	Root surface area	Root volume	Root number	Single root length
Experiment 1 (seedling stage)						
Total nitrogen accumulation	LN	0.96^**^	0.95^**^	0.93^**^	-0.92^*^	0.96^**^
	HN	0.86^*^	0.88^*^	0.64	-0.97^**^	0.93^**^
Net NH_4_^+^ influx	LN	0.93^**^	0.86^*^	0.94^**^	-0.92^**^	0.95^**^
	HN	0.90^*^	0.93^**^	0.76	-0.98^***^	0.96^**^
NH_4_^+^ uptake *via* apoplasmic pathway	LN	0.92^**^	0.86^*^	0.95^**^	-0.96^**^	0.96^**^
	HN	0.55	0.56	0.80	-0.75	0.65
Experiment 2 (heading stage)						
Total nitrogen accumulation at heading stage	LN	0.88^*^	0.91^*^	0.93^**^	-0.89^*^	0.93^**^
	HN	0.10	0.40	0.45	0.69	-0.40
Total nitrogen accumulation at maturity stage	LN	0.88^*^	0.91^*^	0.93^**^	-0.89^*^	0.93^**^
	HN	0.10	0.40	0.45	0.69	-0.40
Grain yield	LN	0.83^*^	0.90^*^	0.97^**^	-0.81^*^	0.87^*^
	HN	0.74	0.87^*^	0.84^*^	-0.26	0.54

HN: high nitrogen application (40 mg L^-1^), LN: low nitrogen application (5 mg L^-1^); *, ** and *** indicate significant correlation at P < 0.05, P < 0.01 and P < 0.001 level, respectively; LN, HN, n=6 (across two varieties and three repeats).

In experiment 2, grain yield was positively and significantly correlated with total root length, root surface area, root volume, and single root length at heading and maturity stages under both HN and LN but not with total root length and single root length under LN, and negatively and significantly with root number under LN (**Table 4**).

### Correlations of nitrogen absorption traits and root anatomical characteristics

In experiment 1, significant and positive correlations were observed among the three nitrogen absorption traits under LN and HN but correlations of NH_4_^+^ uptake *via* apoplasmic pathway with total nitrogen accumulation and net NH_4_^+^ influx under HN (**Table 5**). Living cortical area, stele area, root diameter, stele diameter, and xylem vessel number were positively correlated with the three nitrogen absorption traits under LN and HN, of which most correlations were significant (**Table 5**). The proportion of root aerenchyma was significantly and negatively correlated with the three nitrogen absorption traits under LN and HN but not with NH_4_^+^ uptake *via* apoplasmic pathway under HN (**Table 5**).

**Table 5:** Correlations of root anatomical characteristics at seedling and heading stages with total nitrogen accumulation, net NH_4_^+^ influx, NH_4_^+^ uptake *via* apoplasmic pathway and grain yield.

Traits	Nitrogen treatment	TN	NI	LCA	RCA	SA	RD	SD	XN
Experiment 1 (seedling stage)									
Total nitrogen accumulation	LN	−	0.85^*^	0.97^**^	-0.91^*^	0.96^**^	0.98^***^	0.92^*^	0.93^**^
	HN	−	0.98^***^	0.96^**^	-0.90^*^	0.96^**^	0.95^**^	0.94^**^	0.78
Net NH_4_^+^ influx	LN	0.85^*^	−	0.85^***^	-0.88^***^	0.79^**^	0.81^**^	0.86^***^	0.59^*^
	HN	0.98^***^	−	0.86^***^	-0.70^*^	0.87^***^	0.88^***^	0.89^***^	0.43
NH_4_^+^ uptake *via* apoplasmic pathway	LN	0.91^*^	0.96^***^	0.96^**^	-0.97^*^	0.96^**^	0.94^**^	0.93^**^	0.81^*^
	HN	0.69	0.73	0.74	-0.62	0.67	0.73	0.64	0.89^*^
Experiment 2 (heading stage)									
Total nitrogen accumulation at heading stage	LN	−	−	0.92^**^	-0.80	0.88^*^	0.95^**^	0.86	0.69
	HN	−	−	-0.54	0.41	-0.20	-0.56	-0.07	-0.56
Total nitrogen accumulation at maturity stage	LN	−	−	0.88^*^	-0.96^**^	0.79	0.86^*^	0.86^*^	0.53
	HN	−	−	-0.25	-0.02	0.06	-0.21	0.37	-0.44
Grain yield^AA^	LN	0.92^**^/0.95^**^	−	0.87^*^	-0.94^**^	0.68	0.83^*^	0.76	0.59
	HN	0.16/0.27	−	0.45	-0.75	0.78	0.51	0.72	0.57

HN: high nitrogen application (40 mg L^-1^), LN: low nitrogen application (5 mg L^-1^); LCA: living cortical area, RCA: proportion of root aerenchyma, SA: stele area, RD: root diameter, SD: stele diameter, XN: xylem vessel number; AA indicates the correlation of grain yield with total nitrogen accumulation at heading stage (the former) and at maturity stage (the latter), *, ** and *** indicate significant correlation at P < 0.05, 0.01 and 0.001, respectively; n=12 (across two varieties, three biological repeats, two technical repeat for each biological repeat) for the correlations of net NH_4_^+^ influx with LCA, RCA, SA, RD, SD, and XN, n = 6 (across two varieties, three biological repeats) for the other correlations.

In experiment 2, total nitrogen accumulation at heading and maturity stages was significantly and positively correlated with the six root anatomical traits under LN, no correlations were observed under HN (**Table 5**). Generally, grain yield was positively correlated with total nitrogen accumulation at heading and maturity stage, with the six root anatomical traits but proportion of root aerenchyma (negative correlation) under LN, similar correlations were found under HN but not significance.

### Correlations of expression of *OsAMT* genes with the nitrogen absorption traits

**Figure 3:**
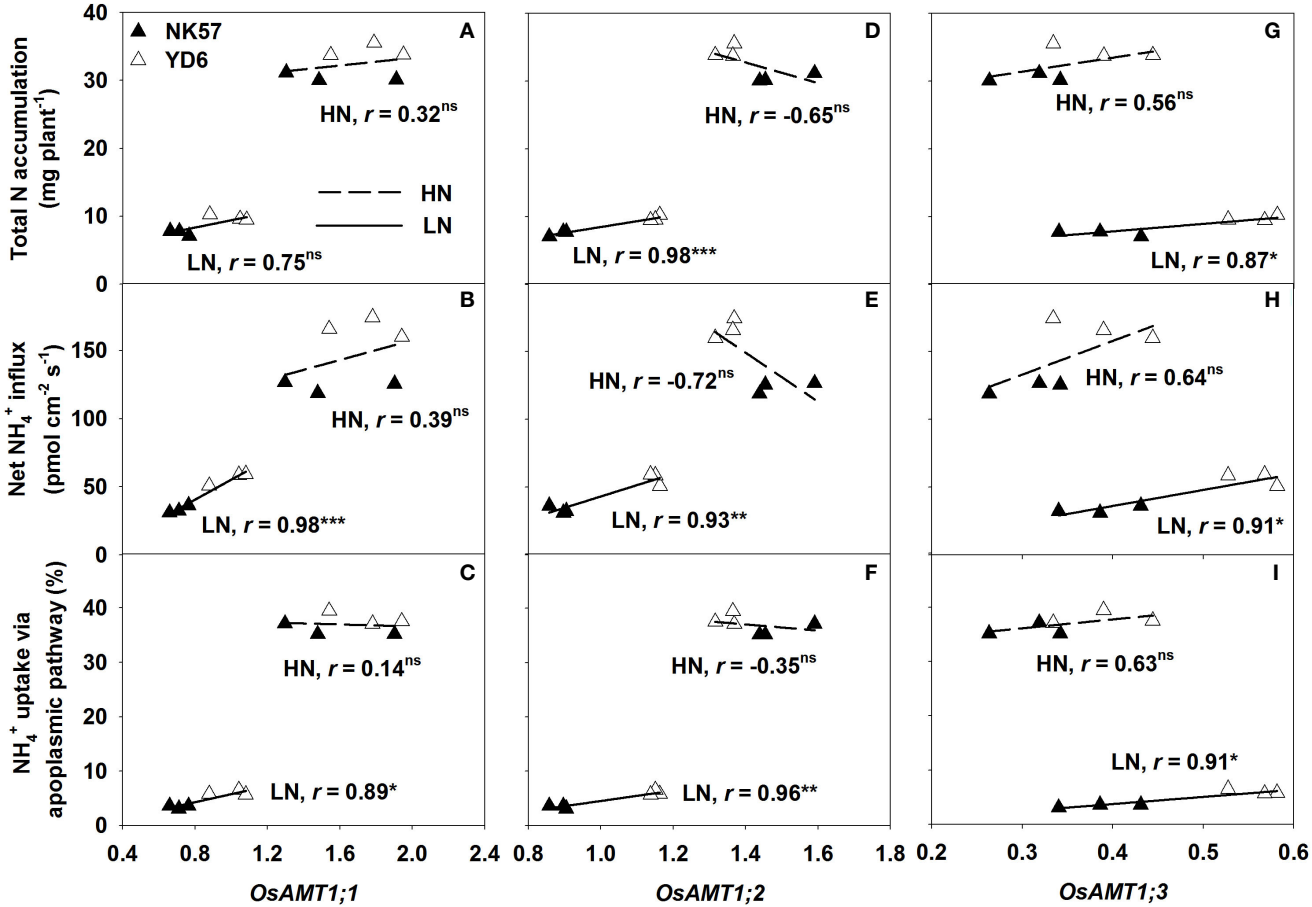
Correlations of the relative expression of *OsAMT1;1* **(A–C)**, *OsAMT1;2* **(D–F)**, *OsAMT1;3* **(G–I)** with the three nitrogen absorption traits under low nitrogen application (LN) and high nitrogen application (HN). *, ** and *** indicate significant correlation at *P* < 0.05, 0.01 and 0.001, ns indicates no significant correlation at *P* < 0.05; n = 6 (across two varieties and three biological repeats).

The expression of *OsAMT1;1*, *OsAMT1;2*, and *OsAMT1;3* genes, which were significantly different between the two varieties, were significantly and positively correlated with the three nitrogen absorption traits under LN (**Figures 3A–I**), however, the correlation between the expression of *OsAMT1;1* and total nitrogen accumulation was insignificant (**Figure 3A**). Nevertheless, the expression of the three genes were unrelated to the three nitrogen absorption traits under HN (**Figures 3A–I**).

## Discussion

In the study, the root morphological and anatomical traits investigated at seedling stage in experiment 1 had significantly positive correlations with these root traits at heading stage in experiment 2 (**Figure S5**), suggesting that rice roots showed consistent growth performance at different growth stages, and as well under different growth conditions (hydroponics and soil culture), the results was consistent with previous reports (Liu et al., 2014; Hou et al., 2019). As known well, field studies of rice roots are difficult and usually imprecise due to the difficulty to extract completely all roots from the field soils; however, Hou et al. (2019) and Price and Tomos (1997) observed that root-related traits of rice plants grown hydroponically were related to water-deficit resistance and nitrogen uptake in the field. Therefore, the high consistency of root performances among hydroponics, soil pot culture, and field planting indicates the availability using rice plants grown hydroponically.

### Large root system and high root length are conductive to nitrogen uptake

In the study, YD6 had larger root system than NK57 at seedling and heading stages (**Figures 1**; **S2**; **Table 2**); accordingly, the variety had higher nitrogen absorption capacity under LN and HN, and a smaller reduction in total nitrogen accumulation (71.6% in YD6 vs.75.2% in NK57) and influx (66.3% vs.73.1%) under LN (**Table 1**). Previous investigations reported that rice varieties with high nitrogen uptake often exhibit large root system, reflected by high root dry weight, total root length, root surface area, and single root length (Ju et al., 2015; Wei et al., 2018; Chen et al., 2020). These results suggest that high root uptake in YD6 was attributed to the large root system, supported by the positive correlation of total nitrogen accumulation with most root traits, especially under LN (**Table 4**). On the other hand, we observed the tight positive association of four root morphological traits with grain yield, especially under LN (**Table 4**). Similarly, Liu et al. (2014) found that genetic improvement significantly increased root dry weight, root length and root surface area, which benefited shoot growth and grain yield in rice varieties. Therefore, for green rice production with reduced nitrogen application, the development of varieties with efficient nitrogen uptake should be focused on improving root system.

### Large root diameter, high stele area, and more xylem vessels are beneficial for nitrogen uptake

YD6 had thicker roots compared to NK57, accompanied by higher nitrogen accumulation and a smaller reduction in total nitrogen accumulation at seedling stage (**Tables 3**; **1**). Similarly, wheat and maize plants with large root diameter had high nitrogen uptake, which accounted for better shoot growth under normal and low nitrogen applications (Yang et al., 2019; Zhang et al., 2020; Schneider et al., 2021). These observations were supported by the positive correlations of root diameter with nitrogen uptake (**Table 5**). Additionally, root diameter-related anatomical characteristics (such as root cortical area, stele area, stele diameter and the number of xylem vessels) were also higher in YD6 than those in NK57 (**Table 3**), and were generally positively correlated with the three nitrogen absorption traits (**Table 5**). In addition, the study observed that tight positive correlations of grain yield with root diameter and stele diameter, especially under LN (**Table 5**). Similarly, compared with old varieties, the modern super rice varieties had higher root diameter, which contributed to their better shoot growth and higher grain yield under same nitrogen application (Liu et al., 2014). Thus, these results suggest that thick roots may be conductive to nitrogen uptake in rice, increasing root diameter should be considered for breeding elite rice varieties suitable for green rice production.

Compared with NK57, YD6 had larger root stele area and more xylem vessels (**Table 3**; **Figure S3**) and smaller reductions in total nitrogen accumulation and NH_4_^+^ influx, and the two traits were positively correlated with the three nitrogen absorption traits, especially under LN (**Table 5**). Similarly, Kadam et al. (2015) and Kong et al. (2017) reported that plant roots with large stele and xylem areas had high water and nutrient transport capacity. Most current maize cultivars have more xylem vessels and are more resistant to low nitrogen stress than earlier variants (York et al., 2015). These results strongly suggest that large root stele area and xylem vessel number may be beneficial for nitrogen uptake in rice, especially under LN. Additionally, our study observed that tight positive correlations of grain yield with these root anatomical characteristics, especially under LN (**Table 5**). Similarly, rice genotypes with larger root stele and root xylem vessels exhibited better growth and higher grain yield (Jeong et al., 2013). Thus, selecting rice varieties with large root stele area and xylem vessel number may also have great significance for green rice production with reduced nitrogen application.

### Response of root characteristics to nitrogen levels and its association with nitrogen uptake and yield formation

It’s noticeable that compared to HN, LN significantly repressed the root growth in two varieties (**Figures 1A–I**; **Table 2**); however, single root length significantly increased in both varieties under LN (**Figures 1E, J**). Therefore, the reduction of total root length, root surface area, and root volume under LN was mainly attributed to the reduction in root number. Similar results were reported in previous studies (Saengwilai et al., 2014; Luo et al., 2022). Noticeably, root number reduced and negatively associated with nitrogen absorption, dry weight and grain yield, especially under LN (**Figure 1**; **Tables 4**; **S6**). Under nitrogen-deficient conditions, less adventitious roots may reduce the competition with each other for nutrients and decrease root metabolic cost, which was beneficial to maintaining growth and development of the whole plant (Giehl and von Wirén, 2014; Saengwilai et al., 2014; Gao et al., 2015). Moreover, genotypes with small root number had more accessible resources to develop longer and deeper roots, and also had higher nitrogen accumulation and grain yield under LN (Saengwilai et al., 2014; Schneider et al., 2021). In line with these observations, the single root length increased under LN, and exhibited favorable association with the three nitrogen absorption traits and grain yield (**Figure 1**; **Table 4**). Previous studies also reported that long and deep root system made it easier to obtain more nitrogen and was conducive to nitrogen accumulation and grain yield formation in rice under LN (Arai-Sanoh et al., 2015; Ju et al., 2015; Chu et al., 2018; Chen et al., 2020). Therefore, these results together suggest that less root number and enhanced root elongation in responses to LN are favorable for nitrogen absorption, developing rice varieties with long and deep roots could promote the coordination of high yield and low nitrogen input for green rice production.

In comparison with HN, LN treatment increased lignin deposition (**Figure S4**). Ren et al. (2015) also reported similar observation, and many genes involved in lignin biosynthesis were upregulated in rice seedlings under nitrogen deficiency (Shao et al., 2020). The three nitrogen absorption characteristics sharply decreased under LN, and were significant correlations with lignin content across the two nitrogen treatments (**Figure S6**). Yang et al. (2022) found that the increased root lignification under a high NH_4_^+^/NO_3_^-^ ratio resulted in poor nitrogen uptake in wheat. For the possible cause, the enhanced root lignin content may incur the formation of root apoplasmic barriers, and inevitably impedes water and nitrogen uptake *via* the apoplasmic pathway (Ren et al., 2015; Huang et al., 2019). Therefore, the elevated root lignin content induced by LN should partially account for the decreases nitrogen uptake in the two varieties.

In the study, LN promoted RCA formation and reduced the living cortical area in both varieties (**Table 3**; **Figure S3**), the observation was consistent with Abiko and Obara (2014). Additionally, RCA was negatively correlated with nitrogen absorption, dry weight, and grain yield, especially under LN (**Table 5**; **Table S7**). The RCA formation reduced the number and area of living cortical cells and seemingly limited nutrient transport *via* the cell-to-cell pathway (Schneider et al., 2017). Additionally, the enhanced RCA formation resulted in long and tortuous routes and fewer intercellular routes, thereby erecting greater effects on the apoplasmic pathway than on the cell-to-cell pathway in maize roots (Hu et al., 2014). Previous study showed that genotypes with high nitrogen use efficiency and yield had less RCA formation compared with ones with low efficiency and yield under LN, and relieved the stress of nitrogen deficit (Yang et al., 2019). These findings imply that the enhanced RCA induced by LN may hinder nitrogen transport *via* the apoplasmic pathway, and may be also responsible for low nitrogen accumulation and low dry matter accumulation and grain yield in the study.

In both varieties, LN significantly decreased NH_4_^+^ uptake *via* the apoplasmic pathway (**Table 1**), and also decreased the expression of *OsAMT1;1*, *OsAMT1;2* genes (**Figure 2A**). Similarly, Sonada et al. (2003) and Li et al. (2016) also reported the down-regulation of the expression of the two genes in rice root epidermis and stele under LN. Yuan et al. (2007) showed that *AMT1;2* mediated NH_4_^+^ uptake *via* apoplasmic pathway. On the other hand, YD6 showed high nitrogen accumulation, high NH_4_^+^ uptake *via* apoplasmic pathway, and high expression of *OsAMT1;1*, *OsAMT1;2*, *OsAMT1;3* genes under LN (**Table 1**; **Figure 2A**), and the expression of the three genes were positively correlated with NH_4_^+^ uptake *via* apoplasmic pathway (**Figures 3C, F, I**). These findings strongly imply that the expression of *OsAMT* genes (especially *OsAMT1;1*, *OsAMT1;2* genes) is tightly associated with NH_4_^+^ uptake transport in the root apoplasmic pathway under LN. Under LN, high expression of *OsAMT* genes enhanced root nitrogen uptake, which further increased leaf photosynthetic rate and promoted grain yield formation in rice (Ranathunge et al., 2014; Ferreira et al., 2015). Therefore, it should be possible approach to develop rice variety with high expression of ammonium transporter for green rice production with nitrogen reducing application.

### Candidate causes for high nitrogen uptake under LN in YD6

**Figure 4:**
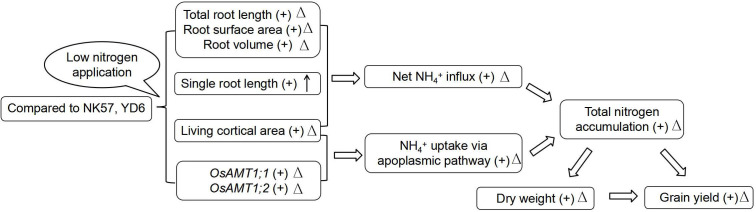
A proposed mechanism for higher nitrogen uptake and higher grain yield in YD6 relatively to NK57 under low nitrogen application. “+” indicates that the trait value of YD6 is higher than that of NK57; “Δ” indicate that the trait value of YD6 has smaller degree of reduction under LN than that of NK57; “↑” indicate that the single root length of YD6 has higher degree of increase under LN than that of NK57.

As compared to NK57, YD6 has higher nitrogen uptake under LN and smaller decrease in total nitrogen accumulation under LN at the three stages (**Table 1**), companying higher net NH_4_^+^ influx and significant positive correlations among the three nitrogen absorption traits at seedling stage, especially under LN (**Tables 1**; **5**; **Figure S1**). Previous research observed the strong linkage between high NH_4_^+^ uptake *via* the apoplasmic pathway and high nitrogen accumulation in plants (Yuan et al., 2007). These results suggest that high NH_4_^+^ uptake *via* the apoplasmic pathway may also partially contribute to high nitrogen uptake in YD6 under LN (**Figure 4**).

YD6 had larger root system relative to NK57, especially under LN (**Figures 1A–J**; **Table 2**), as well as lower reduction degree in total root length, root surface area and root volume at seedling and heading stages) but higher degree increase in single root length. Previous studies also showed that rice varieties with higher nitrogen accumulation had greater the former three root traits under LN than varieties with lower nitrogen accumulation under LN (Ju et al., 2015). These root characteristics were positively correlated with the three nitrogen absorption traits, especially under LN (**Table 4**). Thus, high nitrogen uptake in YD6 should be attributed to the large and deep root system due to the small degree of reduction in root traits under LN (**Figure 4**). In addition, root physiological characteristics such as root vigorous activity (e.g. oxidation activity) and root hormones are closely related to nitrogen uptake. Nitrogen efficient rice varieties with large and deep root also had high root oxidation activity (Ju et al., 2015), vigorous roots produced high contents of cytokinins and indole-3-acetic acid under LN, which were advantageous to delaying plant senescence and maintaining plant growth (Arai-Sanoh et al., 2015; Wu et al., 2019). On the other hand, high contents of cytokinins and indole-3-acetic acid enhanced root nitrogen uptake by altering root system architecture and up-regulating the expression of nitrogen transporter genes under LN (Wu et al., 2019; Luo et al., 2022). Thus, the high nitrogen uptake in YD6 may also be attributed to its high root physiological characteristics, the mechanism remains to be further clarified.

In the study, YD6 had higher expression of root *OsAMT1;1, OsAMT1;2*, *OsAMT1;3* genes than NK57 under LN (**Figure 2A**), and YD6 also had smaller degree reductions in the expression of *OsAMT1;1* and *OsAMT1;2* under LN, but larger increase in *OsAMT1;3* expression (**Figure 2A**). Similarly, Shi et al. (2010) found that rice varieties with high nitrogen uptake exhibited upregulated expression of root *OsAMT1;1* gene under LN, LN boosted the expression of *OsAMT1;3* in low-nitrogen tolerant rice varieties but not in low nitrogen-intolerant rice varieties (Gaur et al., 2012), transgenic rice plants with over-expression of *OsAMT1;3* showed stronger resistance to low nitrogen condition (Ferreira et al., 2015). Additionally, the expression of the three genes was tightly and favorably related with nitrogen uptake under LN (**Figures 3A–I**). The absence of gene *AMT1;2* hindered NH_4_^+^ uptake *via* the apoplasmic pathway in *Arabidopsis* roots (Yuan et al., 2007). Therefore, high expression of *OsAMT* genes should contribute to higher nitrogen uptake in YD6 under LN (**Figures 3A–I**; **4**).

In comparison with NK57, YD6 exhibited lower RCA and high root living cortical area under LN and HN, and had smaller degree decrease in root living cortical area under LN (**Table 3**; **Figure S3**). Additionally, RCA and root living cortical area adversely affected nitrogen uptake, especially under LN (**Table 4**). Yang et al. (2019) reported that maize genotypes exhibiting high tolerance to low-nitrogen application had less RCA under LN, Hu et al. (2014) and Schneider et al. (2017) also reported that larger RCA and less living cortical area may diminish the intercellular routes for nutrient transport *via* the apoplasmic pathway in maize and wheat roots. These results together suggest that high living cortical area may partially explain high nitrogen accumulation in YD6 under LN (**Figure 4**).

### Root size and grain yield formation under LN

Compared to NK57, YD6 had smaller reduction degree in grain yield under LN (**Table 2**), this was also accompanied by smaller reduction degree in total root length, root surface area, root volume (**Figures 1F–H**) and higher increase degree in single root length under LN (**Figure 1J**). Additionally, YD6 maintained higher root living cortical area, root diameter and stele diameter but root number and RCA area under LN compared with NK57 (**Figure 1**; **Table 3**). Similarly, Ju et al. (2015) reported that nitrogen-efficient rice varieties maintained higher root biomass, total root length and deeper roots than nitrogen-inefficient rice varieties under LN, which counted for more grain yield under LN. For possible explanation, deep root system produced high cytokinin flux transported to aboveground plants, which promoted rice grain yield formation under LN (Arai-Sanoh et al., 2015), Wu et al. (2016) found that more root cytokinins from roots to aboveground plants contributed the small reduction of spikelets per panicle under heat stress. In addition, wheat varieties with high grain yield showed smaller reduction degree in root diameter at late growth stage than varieties with low grain yield under LN (Zhang et al., 2020). These results totally suggest that maintaining high performances of several root traits is also beneficial for promoting rice yield formation under LN (**Tables 2**, **3**; **Figures 1**, **4**; **Table S3**), developing varieties with positive root responses to nitrogen reduction should be a considerable target for green rice production in the future.

## Conclusion

Low nitrogen treatment significantly decreased nitrogen uptake (nitrogen accumulation, net NH_4_^+^ influx, and NH_4_^+^ uptake *via* apoplasmic pathway) and grain yield in both rice varieties. Lower NH_4_^+^ uptake *via* the apoplasmic pathway under LN was associated with enhanced lignin deposition in the root cell wall, lower root cortical area and expression of the *OsAMT1;1*, *OsAMT1;2* genes in rice roots. Compared to NK57, YD6 had high nitrogen accumulation and high grain yield under LN, companied with larger root system, high root living cortical area, and elevated expression of the *OsAMT1;1* and *OsAMT1;2* genes; YD6 had smaller reductions in total root dry weight, total root length, root surface area, and root volume, and had larger reduction in root number and larger increase in single root length under LN. These responses of root traits may account for the high nitrogen uptake and grain yield in YD6 plants exposed to LN. The findings suggest that root beneficial plasticity should be prioritized for selecting nitrogen-efficient rice varieties to reduce the usage of nitrogen fertilizers for enabling green rice production.

## Data availability statement

The original contributions presented in the study are included in the article/**Supplementary Material**. Further inquiries can be directed to the corresponding author.

## Author contributions

LL and KC designed the experiments. LL carried out the experiments. KC and LL together analyzed the data and wrote the manuscript. XQ and YW provided experimental assistance. JH and SP gave guidance for experimental design and revised the manuscript. All authors contributed to the article and approved the submitted version.
