# Supplementary material for: Varietal responses of root characteristics to low nitrogen application explain the differing nitrogen uptake and grain yield in two rice varieties
Source: Front Plant Sci. 2023 Aug 3;14:1244281. doi: 10.3389/fpls.2023.1244281 (PMC10435752; doi:10.3389/fpls.2023.1244281)
Supplement: Supplementary file 1 [file DataSheet_1.docx]

**Supplementary Tables (7) and Figures (6)**

**Table S1** The dry matter accumulation in NK57 and YD6 under low and high nitrogen applications at seedling stage and heading and maturity stages

| N treatment | Variety | Stem | Leaf | Panicle | Others |
| --- | --- | --- | --- | --- | --- |
|  |  | g plant^-1^ | g plant^-1^ | g plant^-1^ | g plant^-1^ |
| Experiment 1 |  |  |  |  |  |
| Seedling stage |  |  |  |  |  |
| LN | NK57 | 0.27 b | 0.19 b | － | － |
|  | YD6 | 0.35 b* | 0.27 b* | － | － |
| HN | NK57 | 0.62 a | 0.51 a | － | － |
|  | YD6 | 0.78 a* | 0.65 a* | － | － |
| Experiment 2 |  |  |  |  |  |
| Heading stage |  |  |  |  |  |
| LN | NK57 | 18.11 b | 6.05 b | 3.92 b | － |
|  | YD6 | 24.48 b* | 6.07 b | 4.08 b | － |
| HN | NK57 | 22.96 a | 10.08 a | 5.12 a | － |
|  | YD6 | 29.25 a* | 9.68 a | 5.03 a | － |
| Maturity stage |  |  |  |  |  |
| LN | NK57 | 17.25 b | 2.25 b | － | 2.57 b |
|  | YD6 | 23.67 a | 1.65 b | － | 3.97 a* |
| HN | NK57 | 26.01 a | 5.29 a | － | 4.96 a |
|  | YD6 | 30.45 a* | 4.75 a | － | 4.80 a |

HN: high nitrogen application (40 mg L^-1^), LN: low nitrogen application (5 mg L^-1^); Others: panicle rachis and unfilled grains; different lower-case letters within an identical column indicate significant difference between HN and LN at *P* < 0.05 (LSD test) for the same rice variety; * within an identical column indicates significant difference between YD6 and NK57 at *P* < 0.05 (LSD test) for the given N treatment.

**Table S2** The nitrogen concentrations in NK57 and YD6 under low and high nitrogen applications at seedling and heading and maturity stages

| N treatment | Variety | N_Root_ | N_Stem_ | N_Leaf_ | N_Panicle_ | N_Grain_ | N_Others_ |
| --- | --- | --- | --- | --- | --- | --- | --- |
|  |  | mg g^-1^ | mg g^-1^ | mg g^-1^ | mg g^-1^ | mg g^-1^ | mg g^-1^ |
| Experiment 1 |  |  |  |  |  |  |  |
| Seedling stage |  |  |  |  |  |  |  |
| LN | NK57 | 8.70 b | 9.26 b | 18.09 b | － | － | － |
|  | YD6 | 8.30 b | 8.71 b | 17.73 b | － | － | － |
| HN | NK57 | 13.32 a | 16.68 a | 30.57 a | － | － | － |
|  | YD6 | 11.96 a | 14.84 a | 27.45 a | － | － | － |
| Experiment 2 |  |  |  |  |  |  |  |
| Heading stage |  |  |  |  |  |  |  |
| LN | NK57 | 7.50 b* | 6.03 b | 18.39 b | 12.95 a | － | － |
|  | YD6 | 5.70 a | 5.59 b | 18.17 b | 14.65 b | － | － |
| HN | NK57 | 9.57 a* | 9.74 a | 28.05 a* | 16.70 a | － | － |
|  | YD6 | 6.17 a | 8.66 a | 23.14 a | 16.08 a | － | － |
| Maturity stage |  |  |  |  |  |  |  |
| LN | NK57 | 6.31 a* | 4.67 b | 7.60 b | － | 10.21 b | 11.37 a |
|  | YD6 | 4.50 b | 4.33 b | 6.23 b | － | 11.90 a* | 9.91 a |
| HN | NK57 | 7.43 a* | 7.40 a* | 12.15 a* | － | 13.71 a | 12.00 a |
|  | YD6 | 6.35 a | 6.55 a | 9.67 a | － | 12.74 a | 11.31 a |

HN: high nitrogen application (40 mg L^-1^), LN: low nitrogen application (5 mg L^-1^); N_Root_: root nitrogen concentration; N_stem_: stem nitrogen concentration; N_leaf_: leaf nitrogen concentration; N_panicle_: panicle nitrogen concentration; N_grain_: filled grain nitrogen concentration; N_Others_: nitrogen concentration of unfilled grains and panicle rachis; different lower-case letters within an identical column indicate significant difference between HN and LN at *P* < 0.05 (LSD test) for the same rice variety; * within an identical column indicates significant difference between YD6 and NK57 at *P* < 0.05 (LSD test) for the given N treatment.

**Table S3** Yield components of NK57 and YD6 under under low and high nitrogen applications

| N treatment | Variety | Panicle | Spikelet | TGW | GFP | Biomass | HI |
| --- | --- | --- | --- | --- | --- | --- | --- |
|  |  | No./plant | No./panicle | g | % | g/plant | % |
| LN | NK57 | 11.67 b | 85 b | 24.04 a* | 68.1 a | 38.3 b | 42.3 a |
|  | YD6 | 10.78 b | 116 a* | 22.98 a | 65.6 a | 48.1 b* | 39.1 a |
| HN | NK57 | 15.11 a | 96 a | 24.59 a | 65.1 a | 59.4 a | 38.9 b |
|  | YD6 | 13.33 a | 120 a* | 22.56 a | 68.1 a | 64.6 a | 38.1 a |

TGW: thousand grain weight; GFP: grain filling percentage; HI: harvest index. Different lower-case letters within an identical column indicate significant difference between HN and LN at *P* < 0.05 (LSD test) for the same rice variety; * within an identical column indicates significant difference between YD6 and NK57 at *P* < 0.05 (LSD test) for the given N treatment.

**Table S4** The transpiration stream (TS), transpiration stream concentration factors (TSCF) for PTS and NH_4_^+^ under in YD6 and NK57 under low and high nitrogen applications at seedling stage (experimental 1)

| Nitrogen treatment | Variety | PTS uptake | NH_4_^+^ uptake | TS | PTS in TS | NH_4_^+^ in TS | TSCF for PTS | TSCF for NH_4_^+^ |
| --- | --- | --- | --- | --- | --- | --- | --- | --- |
|  |  | ×10^-1^  mg h^-1^ | mg h^-1^ | ml h^-1^ | ×10^-2^  mg ml^-1^ | ×10^-2^  mg ml^-1^ | ×10^-3^ | ×10^-3^ |
| LN | NK57 | 0.08 b | 0.12 b | 4.13 b | 0.19 b | 3.00 b | 0.20 b | 5.88 a |
|  | YD6 | 0.20 b* | 0.18 b* | 5.04 b* | 0.40 b* | 3.58 b | 0.42 b* | 7.04 a |
| HN | NK57 | 0.25 a | 0.30 a | 5.91 a | 0.42 a | 5.04 a | 0.44 a | 1.24 b |
|  | YD6 | 0.35 a* | 0.40 a* | 7.09 a* | 0.49 a* | 5.60 a | 0.52 a* | 1.38 b |

HN: high nitrogen application (40 mg L^-1^), LN: low nitrogen application (5 mg L^-1^); different lower-case letters within an identical column indicate significant difference between HN and LN at *P* < 0.05 (LSD test) for the same rice variety; * within an identical column indicates significant difference between YD6 and NK57 at *P* < 0.05 (LSD test) for the given nitrogen treatment.

**Table S5** Primers used in this study for qPCR

| Gene | Accession number (NCBI) | Forward sequence (5'-3') | Reverse sequence (5'-3') |
| --- | --- | --- | --- |
| *OsAMT1;1* | AF289477 | TTTTGCTGGGCTTCTCTTGT | ACCATTCCACCACACCCTTA |
| *OsAMT1;2* | AF289478 | CTTCATCGGGAAGCAGTTCT | TGAGGAAGGCGGAGTAGATG |
| *OsAMT1;3* | AF289478 | CGGCTTCGACTACAGCTTCT | GACCAGATCCAGTGGGACAC |
| *OsAMT2;1* | AB051864 | CTGGCTCCTCCTCTCCTACA | CAGGATGTTGTTCGGTGAGA |
| *OsAMT2;2* | AP003252 | GCCTCGACGTCATCTTCTTC | TTGTGGAGGATCATCATGGA |
| *OsAMT2;3* | AP003252 | GCCTCGACGTCATCTTCTTC | GGAAGGTGGATTTCTTGTGC |
| *OsAMT3;1* | AB083582 | ACCAAGGACAGGGAGAGGTT | AAGATGACGTCGAGGCAAGT |
| *OsAMT3;2* | AC104487 | GCACAGAAGGACAGGGAGAG | GCAGATGTTGGTGTTGAGGA |
| *OsAMT3;3* | AP004775 | CGAGCATCACCATCATCATC | ATGACACCCCACTGGAAGAG |
| *OsAMT4* | AC091811 | CTGGCCTCAAGAAGATGGACA | AGCTGCTTCACGTACTTGATCG |
| *Actin* | AB047313 | ATGAAGATCAAGGTGGTCGC | GATCTCAGCCTTGGCAATCC |

**Table S6** Correlations of root morphological characteristics at seedling and heading stages) with total dry weight

| Traits | Nitrogen treatment | Total root length | Root surface area | Root volume | Root number | Single root length |
| --- | --- | --- | --- | --- | --- | --- |
| Experiment 1  (seedling stage) |  |  |  |  |  |  |
| Total dry weight | LN | 0.96^**^ | 0.93^**^ | 0.91^*^ | -0.90^*^ | 0.95^**^ |
|  | HN | 0.88^*^ | 0.89^*^ | 0.87^*^ | -0.96^**^ | 0.93^**^ |
| Experiment 2  (heading stage) |  |  |  |  |  |  |
| Total dry weight at heading stage | LN | 0.93^**^ | 0.91^*^ | 0.81 | -0.87^*^ | 0.97^**^ |
|  | HN | 0.95^**^ | 0.88^*^ | 0.53 | -0.66 | 0.88^*^ |
| Total dry weight at maturity stage | LN | 0.87^*^ | 0.91^*^ | 0.93^**^ | -0.83 | 0.91^*^ |
|  | HN | 0.89^*^ | 0.94^*^ | 0.75 | -0.47 | 0.74 |

HN: high nitrogen application (40 mg L^-1^), LN: low nitrogen application (5 mg L^-1^); * and ** indicate significant correlation at *P* < 0.05 and *P* < 0.01 level, respectively; LN, HN, n=6 (across two varieties and three repeats).

**Table S7** Correlations of root anatomical characteristics at seedling and heading stages with total dry weight

| Traits | Nitrogen treatment | LCA | RCA | SA | RD | SD | XN |
| --- | --- | --- | --- | --- | --- | --- | --- |
| Experiment 1  (seedling stage) |  |  |  |  |  |  |  |
| Total dry weight | LN | 0.96^**^ | -0.90^*^ | 0.94^**^ | 0.98^***^ | 0.91^*^ | 0.95^**^ |
|  | HN | 0.97^**^ | -0.90^*^ | 0.93^**^ | 0.97^**^ | 0.93^**^ | 0.95^**^ |
| Experiment 2  (heading stage) |  |  |  |  |  |  |  |
| Total dry weight at heading stage | LN | 0.99^***^ | -0.91^*^ | 0.90^*^ | 0.99^***^ | 0.90^*^ | 0.77 |
|  | HN | 0.87^*^ | -0.93^**^ | 0.66 | 0.88^*^ | 0.80^*^ | 0.84^*^ |
| Total dry weight at maturity stage | LN | 0.91^*^ | -0.92^**^ | 0.69 | 0.87^*^ | 0.75 | 0.64 |
|  | HN | 0.69 | -0.87^*^ | 0.76 | 0.72 | 0.80 | 0.74 |

HN: high nitrogen application (40 mg L^-1^), LN: low nitrogen application (5 mg L^-1^); LCA: living cortical area, RCA: proportion of root aerenchyma, SA: stele area, RD: root diameter, SD: stele diameter, XN: xylem vessel number; *, ** and *** indicate significant correlation at *P* < 0.05, 0.01 and 0.001, respectively; LN, HN, n=6 (across two varieties and three repeats).

**Figure legends**


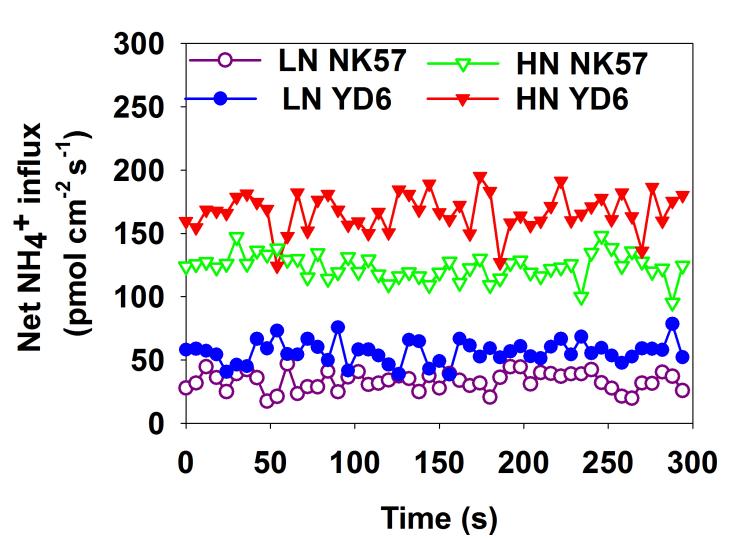


**Fig. S1** The net NH_4_^+^ influx on the root surface in YD6 and NK57 under low and high nitrogen applications at seedling stage (experimental 1)

HN: high nitrogen application (40 mg L^-1^), LN: low nitrogen application (5 mg L^-1^); each point is shown as mean (n=6).


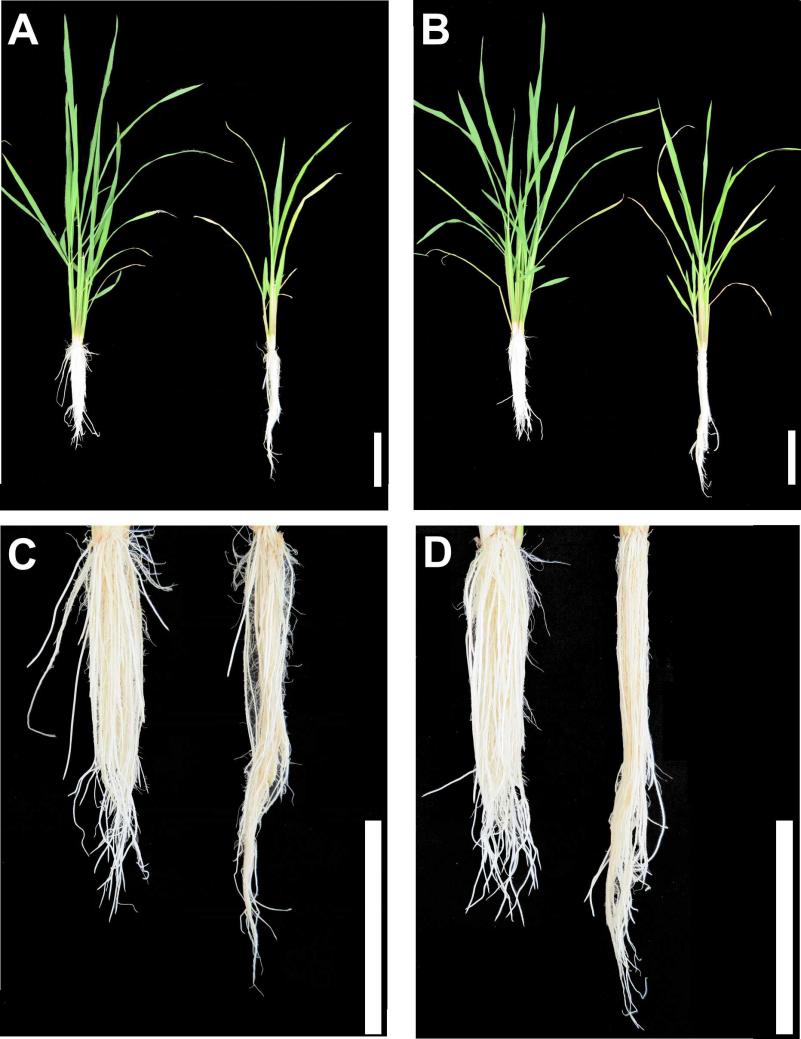


**Fig. S2** The rice plants under low and high nitrogen applications at seedling stage (experimental 1)

HN: high nitrogen application (40 mg L^-1^), LN: low nitrogen application (5 mg L^-1^); A: NK57 under HN and LN; B: YD6 under HN and LN, C: NK57 under HN and LN; D: YD6 under HN and LN, scales= 10 cm.


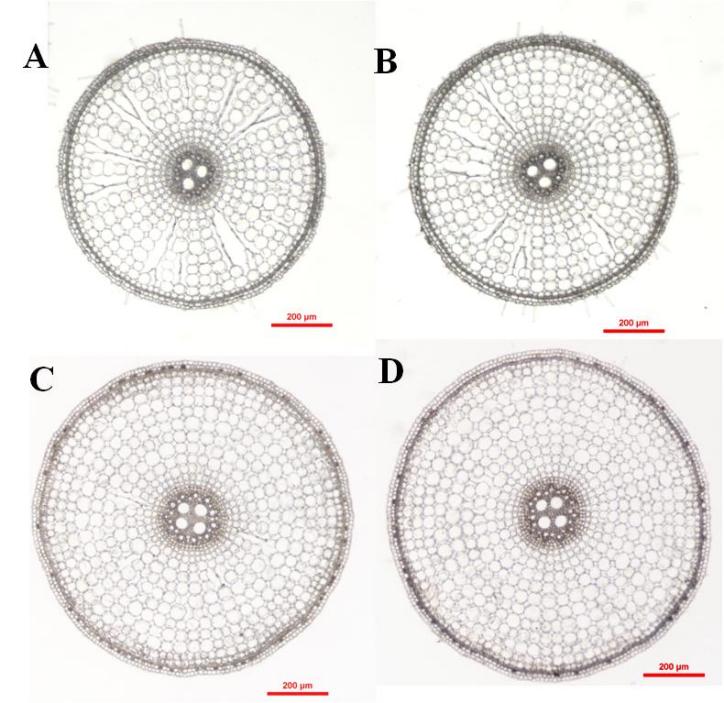


**Fig. S3** The root anatomical characteristics in YD6 and NK57 under low and high nitrogen applications at seedling stage (experimental 1)

HN: high nitrogen application (40 mg L^-1^), LN: low nitrogen application (5 mg L^-1^); A: NK57 under LN; B: NK57 under HN, C: YD6 under LN; D: YD6 under HN, scales= 200 μm.


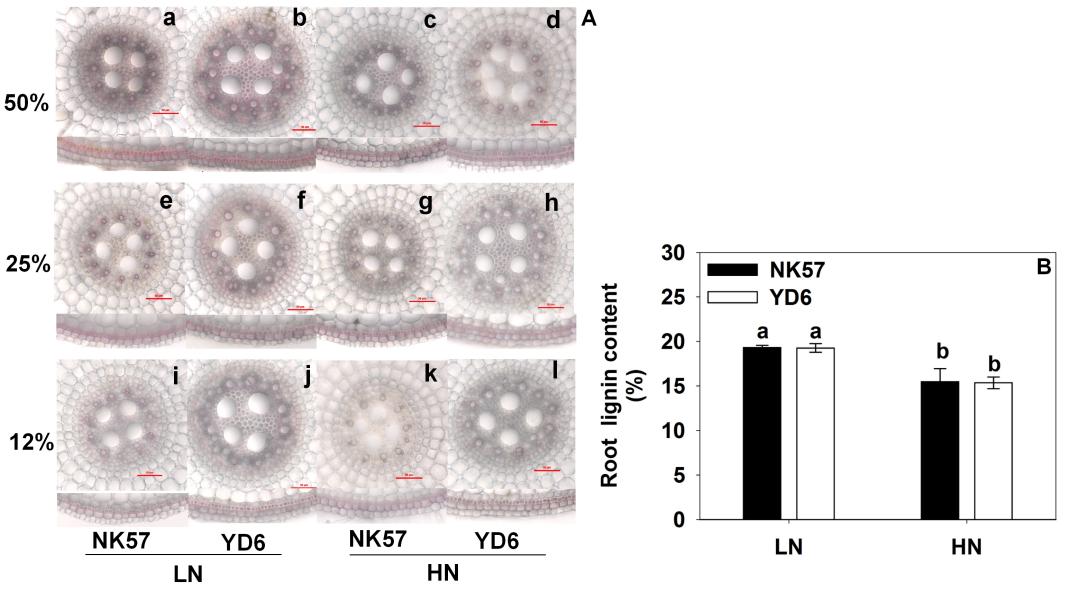


**Fig. S4** The root lignin staining (red) (A) and root lignin content (B) in YD6 and NK57 under low and high nitrogen applications at seedling stage (experimental 1)

HN: high nitrogen application (40 mg L^-1^), LN: low nitrogen application (5 mg L^-1^); a, e, i, b f, j: root stele and sclerenchyma at 50%, 25%, 12% distance from root tip of adventitious roots under LN, respectively; c, g, k, d, h, l: root stele and sclerenchyma at 50%, 25%, 12% distance from root tip of adventitious roots under HN, respectively; different lower-case letters on top of histograms denote significant difference between HN and LN at *P* < 0.05 (LSD test) for the same rice variety.


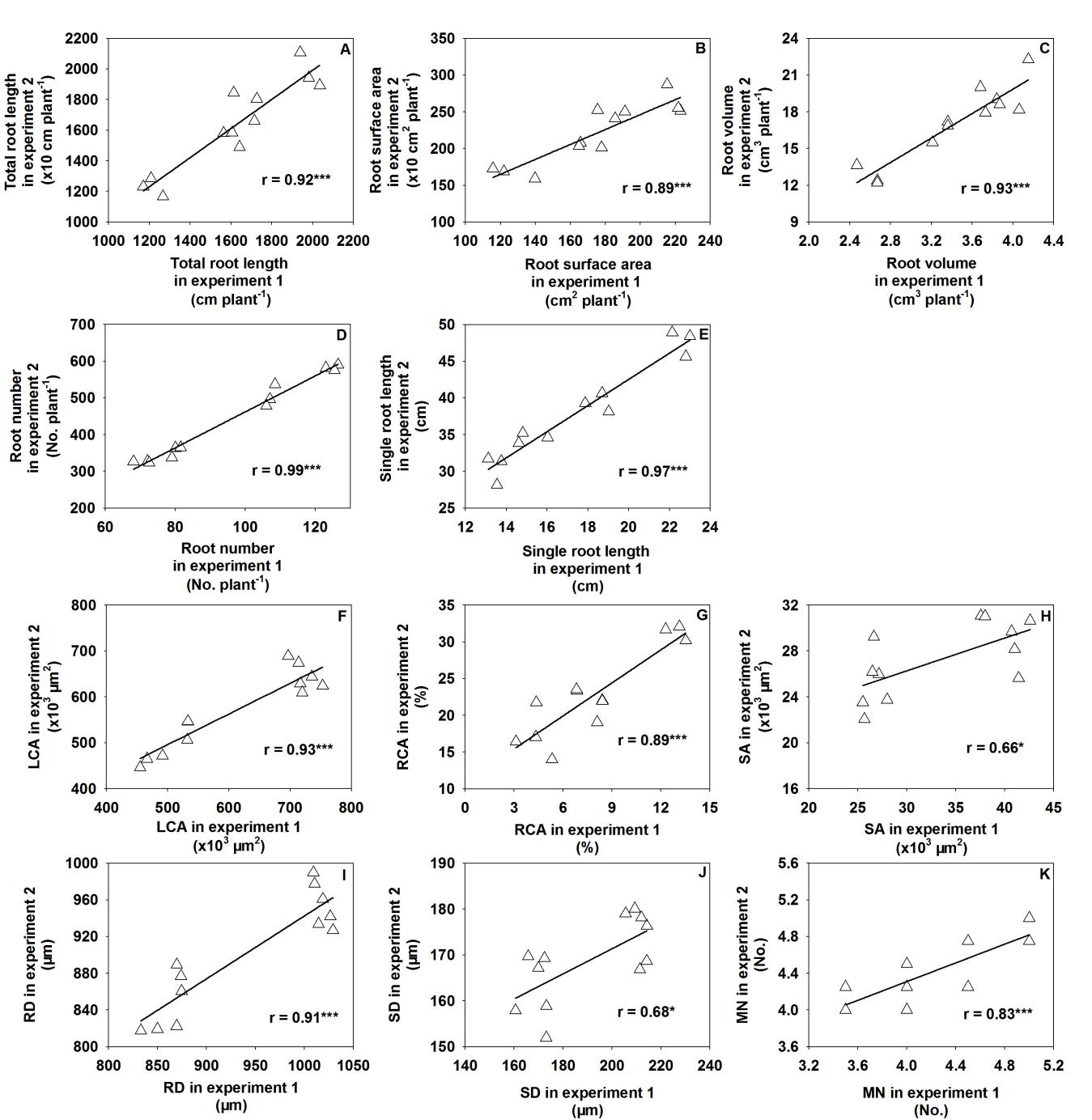


**Fig. S5** The correlations of the root morphological and anatomical characteristics at seedling stage with these root characteristics .at heading and maturity stages

* and *** indicate significant correlation at *P* < 0.05 and 0.001, respectively; n = 12 (across two varieties, two nitrogen treatments, three biological repeats); LCA: living cortical area, RCA (%): the proportion of root aerenchyma, SA: stele area, RD: root diameter, SD: stele diameter, XN: number of xylem vessels, CCFN: cortical cell file number.


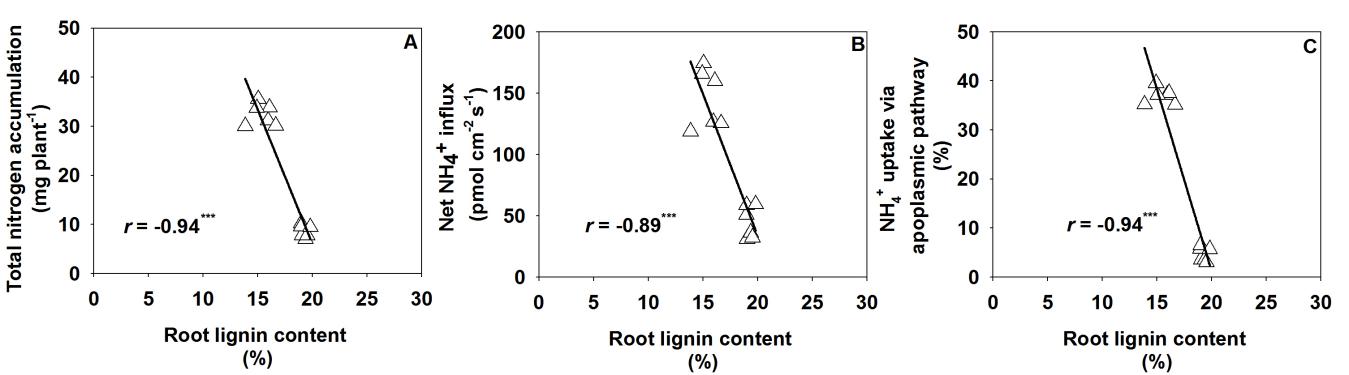


**Fig. S6** Correlations of root root lignin content with total nitrogen accumulation (A), net NH_4_^+^ influx (B), and NH_4_^+^ uptake via apoplasmic pathway (C) at seedling stage

*** indicates significant correlation at *P* < 0.001; n = 12 (across two varieties, two nitrogen treatments, three biological repeats).
